# Online learning during the COVID-19 pandemic: the wellbeing of Chinese migrant children—a case study in Shanghai

**DOI:** 10.3389/fpsyg.2024.1332800

**Published:** 2024-01-29

**Authors:** Qifan Ding, Qiaobing Wu, Qi Zhou

**Affiliations:** Department of Applied Social Sciences, The Hong Kong Polytechnic University, Kowloon, Hong Kong SAR, China

**Keywords:** migrant children, COVID-19, online learning, emotional wellbeing, social wellbeing, physical wellbeing

## Abstract

**Introduction:**

This study uses Bronfenbrenner’s bioecological model as its theoretical framework to consider the findings of an investigation of the emotional, social, and physical wellbeing of Chinese migrant children and lessons learned from the COVID-19 pandemic in urban areas. This study expands our perspective by combining the views of students, parents, and teachers to explore the emotional, social and physical wellbeing of migrant children in Shanghai who were participating in online learning during the COVID-19.

**Methods:**

Observation and semi-structured interviews were carried out to collect data for this case study. Thirty-one migrant children, nine parents, 10 teachers and a school principal from a Shanghai junior high school participated in this research. Qualitative data were analyzed using thematic analysis.

**Results and Discussion:**

The findings indicated that although video-recorded lessons were high quality, it put pressure on migrant children due to the lessons containing only new material with no reviews and reduced opportunities for them to interact with their own teachers. In addition, the differences in study progress between the migrant children and the local children that showed up during the online learning, and neglect from teachers and policymakers, made the migrant children anxious, angry and confused about their future. Besides, parents install monitors at home to support their children’s online learning, but it had the opposite effect and simply provoked increased conflicts between children and their parents. Finally, although the online lessons have affected the optical health of students, the subsequent additional cooking lessons have mitigated the optical health problem and strengthened the connections between home and school.

**Conclusion:**

The inequalities of education encountered by migrant children during the COVID-19 period have made them realize the disparities they have suffered in Shanghai. The exposure of this problem raises the prospect of a reform of educational policies for migrant children in the future.

## 1 Introduction

The COVID-19 pandemic has been an international public emergency since 2020, and the public health response to the lockdowns, social distancing, school closures and economic shutdowns have serious impacts on all aspects of children and adolescents’ development in the world, including physical, psychosocial, and mental health (Tso et al., 2020). Previous studies have found an increased probability of psychological problems in children and adolescents after the COVID-19 pandemic. Children and adolescents experience changes in lifestyle and learning styles, such as school absence due to pandemic preparedness measures, home quarantine, and online learning; these can affect their wellbeing (Fore, 2020; Miao et al., 2023). Concerns were also raised about the availability of sufficient support for vulnerable groups, such as children with learning difficulties and mental health needs. Social distancing regulations across the world have resulted in millions of children being suddenly disconnected from face-to-face education (United Nations Educational, Scientific and Cultural Organization, 2020). Schools and universities have moved their teaching activities online in response to the situation. The United Nations estimated that approximately 463 million children were cut off from their education because they could not access online learning (United Nations Children’s Fund, 2020). It has been discovered that homeschooling was difficult for children from low-income families. A study in the UK revealed that parents experience increased stress as they have to balance childcare, homeschooling and working during the COVID-19 period. The economic impact of the COVID-19 pandemic has also added financial burdens to many families and increased stress on parents. School closures can have an even greater impact on children and young people with mental health needs because they lack the resources that schools would normally provide (Tso et al., 2020).

In 2020, school shutdowns in 188 countries, affecting more than 1.5 billion students in 2020 (Organization for Economic Co-operation and Development, 2021). Traditional face-to-face schools were forced to transform into virtual schools for students with continuing education. Therefore, students were forced to adapt to online learning. At the same time, video conferences and social media become the main venues for knowledge delivery and communication. However, K-12 students lack online learning experience. Even in countries where online learning is developing significantly, such as the United States and Canada, less than 10 per cent of K-12 students have had experience in online learning. Current studies discovered several major concerns about children’s online learning during the COVID-19 pandemic, including internet connection issues, IT facilities issues, reduced learning motivation, students’ adaptability to online learning, eyestrain caused by long staring at screens, insufficient communication with teachers (Bączek et al., 2021; Yan et al., 2021). In addition, online learning increased the socioeconomic inequalities in accessing technological resources. It has been found that children from low-income families have less access to learning materials (computers and textbooks) and effective learning environments (overcrowded households and no access to electricity and the internet) than high-income families. These situations are much more common among children in rural and remote areas. Moreover, several impacts of online learning on children’s wellbeing have also been identified, including overuse of electronic devices, less interaction with classmates and teachers, attention deficits, stress, and depression (Xie et al., 2020).

The Chinese central government also implemented strict quarantine regulations: “School is suspended, but learning continues.” The Ministry of Education of the People’s Republic of China passed a regulation that all schools should stop face-to-face teaching, but all teaching activities should be moved online (Ministry of Education of the People’s Republic of China, 2020a). In China, more than 220 million children and adolescents, including 180 million primary and secondary school students, were confined to their homes (Wang et al., 2020). During this challenging time, the family became a learning space in which parents and caregivers acted as guides to support their children’s study at home. A growing amount of literature has argued that migration has immediate consequences for a child’s wellbeing (Liang, 2016; Li and Jiang, 2018; Xu et al., 2018). For example, studies have shown that migrant children are facing educational inequality in urban China (Ma and Wu, 2019). In China, the term “migrant workers” refers to rural household (hukou) residents who have lived in cities for at least 6 months without holding a local household registration. Children of migrants such as those who move to cities with their migrant parents receive compulsory education (Grade 1 to Grade 9) in the city while maintaining their rural household registration (Ministry of Education of the People’s Republic of China, 2016). In 2021, the migrant population exceeded 375 million (National Bureau of Statistics, 2021). As of the year 2020, around 14.26 million migrant children were eligible for compulsory education when they reached school age (Ministry of Education of the People’s Republic of China, 2020b). Although they live and study in urban cities with their parents, their rural hukou cannot ensure they can get equal access to senior high school education, as local children do. As migrant children grow up, their education needs become increasing intense after they finish compulsory education (Grade 1–Grade 9). In China, students who finished compulsory education should take the Senior High School Entrance Examination. This examination is a national highly competitive examination held in different provinces to grade students who have finished their compulsory education and see if they are qualified to receive senior high school education. In the current Chinese education system, secondary vocational schools and senior high schools are mainly oriented toward the job market and higher education, respectively. Having a university degree is favorable for an individuals’ upward mobility. Migrant children and parents must satisfy the conditions required by the cities they moved to in order for the children to attend the senior high school entrance examination and study in senior high schools, otherwise they have to study in vocational schools. However, vocational education in cities serves to train and produce workers (Shi, 2017). A policy for migrant children’s senior high school entrance examinations was determined by 27 provincial governments in 2014 (Guangming Daily, 2014). Nevertheless, there are huge regional differences in the requirements for allowing migrant children’s participation in the senior high school entrance examination. Those policies only clarify the entrance threshold and screening mechanism rather than increasing the migrant children’s opportunities to get access to educational resources. The entry threshold for senior high schools in some metropolises, such as Shanghai is still high for migrant children.

Although there are many studies on the impact of the COVID-19 pandemic on children’s wellbeing, the wellbeing of migrant children, a vulnerable group in China, was even more neglected in the context of COVID-19-influenced online classes (Fore, 2020; Tso et al., 2020; Abdo Ahmad et al., 2023; Dvorsky et al., 2023; Miao et al., 2023; Pampati et al., 2023). Furthermore, under the COVID-19 context, the successful transition from the traditional education environment to an online teaching-learning one was not an overnight event, as it faced various obstacles and challenges during this period (Crawford et al., 2020). Migrant children in China live in a different social and cultural context compared to general children. In particular, the educational and social systems for migrant populations are different from those in other countries (Shi, 2017; Xu et al., 2018; Han et al., 2020). Chinese migrant children are involved in household registration and the allocation of educational resources, which are closely related to local policies. Findings from previous studies in other countries may not be simply transferred to Chinese migrant children (Exenberger et al., 2019; Abdo Ahmad et al., 2023). In particular, problems migrant children faced in online learning were influenced by local policies, online schooling environments, parents, peers, and teachers compared to the general students. Therefore, this study utilizes Bronfenbrenner’s bioecological model to provide a holistic perspective where all levels from microsystem to macrosystem can be considered. The aim of this paper is to explore how the online learning experience influenced migrant children’s wellbeing in Shanghai, which offers significant insights applicable to post-pandemic education.

## 2 Literature review

To provide a solid foundation for the study, the following literature review was undertaken from several aspects covering the strengths and weaknesses in online learning, roles of parents and schools in online learning and children’s wellbeing during the COVID-19 pandemic.

### 2.1 Strengths and weakness in online learning during the COVID-19

A substantial amount of literature has been written about the benefits and challenges of online learning. The rise of online learning can be attributed to its convenience in terms of location and time. Additionally, online learning offers a cost-effective means of accessing a wider range of information (Yılmaz, 2019; Kim, 2020). As an alternative to traditional learning, it provides students and teachers with both asynchronous and synchronous tools, such as email, chat platforms, and video conferences (Dhawan, 2020). This mode of learning eliminates the need for in-person meetings. Particularly during the COVID-19 pandemic, online learning became a crucial method for maintaining teaching and learning activities in educational institutions.

However, online learning also has some disadvantages that must be considered. Firstly, children typically have low levels of self-control and are prone to being easily distracted or losing concentration, which can negatively impact their ability to learn (Coman et al., 2020). Therefore, adult participation and supervision is necessary for successful online learning (Youn et al., 2012). Second, online learning may not provide enough time and opportunities for children who need more interaction and activities to help them concentrate on learning (Crawford et al., 2020). Thirdly, the quality of online learning platforms is a crucial factor in the development of online learning, as inadequate tools can hinder children’s learning progress. For instance, certain platforms may lack essential features, such as online chat, material sharing, and screen sharing, which can negatively impact learning outcomes. Additionally, online communication is often limited to classmates, offering no opportunity for real-time knowledge and information sharing with teachers. Lastly, online learning may also have negative effects on physical health, as prolonged screen time without any outdoor activity can lead to increased levels of stress and vision problems for both teachers and students (Coman et al., 2020).

Apart from these general impacts of online learning, a systematic review discovered online learning during the COVID-19 period had a negative impact on children’s academic performance in mathematics, reading, language and spelling, and biology. Several factors contribute to this negative impact, such as socio-economic status (family type and family income), access to technology, the learning environment, the quality of online class resources, and feedback from teachers (Cortés-Albornoz et al., 2023). Dvorsky et al. (2023) summarized challenges faced by children and adolescents with special educational needs and dis/abilities during the COVID-19 period. Regarding school supports and services, individualized interventions for academics and behavioral supports, school-based health and mental health services have been discontinued. Regarding school and community environment, it is difficult for those youth to access to digital course materials when using different platforms. They also have limited access to health and mental health services out of school. These pandemic-driven disparities reinforce existing inequalities among students with special educational needs. Guo and Wan (2022) argued this could be described as the digital divide which means the distribution of information and communication technology resources replicates the inequalities inherent in the social structure. Although online learning ensured learning continuity, it added inequality to education inequality to children from different social backgrounds. Disadvantaged groups may not have access to online learning tools and materials. In addition, without face-to-face instruction and parental guidance, they may be unprepared for online learning (Sahlberg, 2020; Guo and Wan, 2022).

### 2.2 The role of parents and schools in online learning

Many studies have found that in online learning environments, the parents of the children take on the following roles: regulators of their child’s online activities, learning coaches or co-educators, and providers of caring relationships (Rice and Dawley, 2009; Waters and Leong, 2014; Nouwen and Zaman, 2018; Borup et al., 2020). These roles reflect research on parental roles prior to the occurrence of the pandemic. Preliminary research also indicates that during COVID-19, parents have taken on these same types of roles during this crisis: regulators, co-educators and emotional supporters.

As regulators, parents play a mediating role to prevent harm and regulate the children’s online activities, doing such things as setting technology use rules for their children and monitoring the use of the equipment (Nouwen and Zaman, 2018). For co-educators, learning activities have moved from school to home during the lockdown period. Physical distancing denied access to sufficient schooling and community support. Children naturally sought help from their parents during such a stressful and turbulent period. Therefore, parents needed to engage in some activities that are normally supposed to be the responsibility of the teachers. They managed to promote the students’ learning by applying instruction, questioning, and listening to feedback (Bhamani et al., 2020). However, parents and caregivers cannot replace the role and function of the teacher in school. Some parents have to spend a lot of time managing their children’s behavior at home. This is especially difficult for parents with a low educational level. COVID-19 forced family education to be incorporated in school education, which has had a huge impact on family and children’s wellbeing. It also discovered that providing time and space to promote teacher-student and parent–child relationships can strengthen the connections between schools, families, and students during the at home online learning period in China (Zhou et al., 2020). In this situation, parents act as a co-educator to cooperate with schools to understand their children’s difficulties. With regard to providing emotional support, parent–child cooperation was stimulated by learning activities during the COVID-19 period. It has been argued that the more time parents spend with their child/children, the better their relationship becomes (Bhamani et al., 2020). In this circumstance, parents should interact with their child/children in order to reduce anxiety and worries.

Besides, when schoolteachers act as educators and provide mental health support, they not only fulfill the role of helping students’ acquire knowledge, but also promote students’ wellbeing (Catalano et al., 2003; Zins et al., 2007). In terms of education, teaching activities did not stop during COVID-19, and many countries were continuing teaching activities based on their own circumstances. It has been found that academic staff, technicians and students in China worked together to continue teaching and learning activities at universities during the lockdown period (Zhang et al., 2020). Australian schools used text messages, phone calls or skype to contact students and parents to identify the needs of the students and to keep track of their learning (Brown et al., 2020). Regarding mental health support, the UNESCO Chair on Health Education and Sustainable Development emphasized that education is a crucial component of health. Schools also play an active role in promoting health-conscious behavior among students. Within the school environment, the promotion of wellbeing through meaningful and transformative learning can serve as a catalyst for the development of a knowledge-based health culture, having far-reaching effects not only on the behaviors of students, but also on those of their families and communities (Colao et al., 2020).

### 2.3 Children’s wellbeing during the COVID-19 pandemic

Children are physiologically undeveloped and may experience negative psychological symptoms when exposed to the unexpected COVID-19 pandemic. Less interaction with peers in school, extended screen time, straining relationships due to extended parental supervision, adversely affecting their wellbeing such as depressive symptoms and anxiety (Miao et al., 2023). A recent study from Canada also revealed that parents with university degrees and who work from home have lower levels of concern about their children’s wellbeing. Parents who have children with disabilities, children of ethnic minorities, children of immigrants can make parents worried about their children’s wellbeing (Abdo Ahmad et al., 2023). It has also been argued that the wellbeing of caregivers directly affects children’s wellbeing (Newland, 2015; Pampati et al., 2023). High levels of parental stress were associated with increased mental health symptoms in children, emphasizing the impact on family dynamics, caregiver burden and parent–child relationships. Existing studies discovered that parents worried about their child/children’s daily social, physical and emotional health (Dong et al., 2020; Doll et al., 2022). Therefore, it has been suggested that it is important to investigate the social, physical and emotional wellbeing of children during the COVID-19 pandemic period (Goldschmidt, 2020).

Social wellbeing refers to individuals being able to build and maintain healthy relationships and interactions with other individuals in their immediate environment. Play is an essential part of children’s social and physical development. During the COVID-19 lockdown, people were confined to their homes and children had fewer opportunities to play outside or see their friends (Goldschmidt, 2020). Peer interaction decreased and parents expressed the concern that their child/children’s social development would be affected. Unfortunately, some parents have noticed changes in their children’s behavior, such as symptoms of sadness, depression and loneliness (Lee et al., 2021). Although students working online have limited contact with peers and no opportunity to have face to face contact with them, the positive interactions that do occur between caregivers and children are healthy. It is good when families can spend more time together. When time is available, caregivers are encouraged to socialize with children by playing games, doing arts and crafts, and listening to music, etc. Caregivers can also spend time with children by watching TV programs together or using educational apps.

Physical wellbeing encompasses the capacity to engage in physical activities and fulfill social roles without being impeded by physical limitations or experiences of bodily pain, as well as the presence of positive biological health indicators (Capio et al., 2014). A systematic review of intervention studies has shown that exercise and physical activity have a positive impact on physical wellbeing (Penedo and Dahn, 2005). In children, physical wellbeing is defined as the ability to participate fully in developmentally appropriate activities in a normal manner. Factors that are crucial to children’s physical wellbeing include nutrition, a clean and safe environment, health care, mental stimulation and access to nurturing relationships (Curtis et al., 2004). During the COVID-19, some parents mentioned that their child/children spent 3 to 4 h per day using a computer in online learning and spend less time doing outdoor activities, thus raising worries among parents about this having a negative impact on child/children’s eyesight (Christensen and Alexander, 2020; Zhao et al., 2020).

Emotional wellbeing is an important dimension in mental health. It comprises happiness and overall life satisfaction. Accordingly, supporting emotional wellbeing is an important responsibility in different areas such as schools and among different groups such as children, adolescents and women (Ross et al., 2020). The strengths coming from emotional wellbeing serve as a buffer against misfortune and mental health issues, and they may be the key to building resilience. During the COVID-19 lockdown period, regarding child/children’s emotions, parents reported that their child/children had increased levels of worry, irritability and anxiety due to social isolation, lack of interaction, and delays in receiving substantive feedback from teachers about their schoolwork. Parents also felt that without formal learning structures and routines being present in online learning environments, their child/children would not develop appropriate self-regulation skills and study habits (Bhamani et al., 2020; Dong et al., 2020).

### 2.4 The ecological systems theory

**Figure 1:**
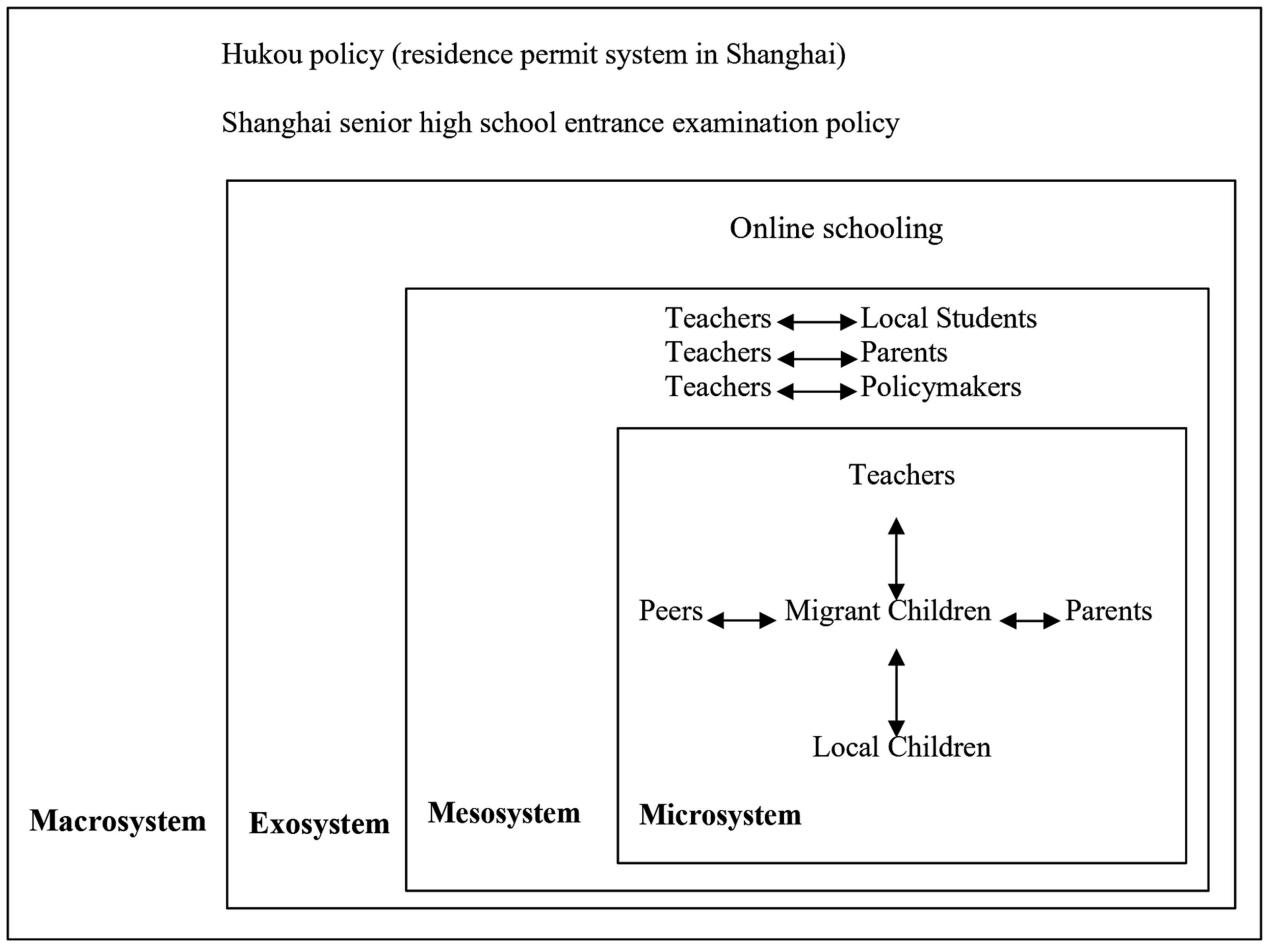
Analytical framework.

The present study uses Bronfenbrenner’s bioecological model as the theoretical framework for considering the findings regarding the wellbeing of migrant children, and lessons learned from the COVID-19 pandemic pertaining to secondary school education, especially in the case of migrant children in the cities. Fernandes et al. (2012) emphasized that children’s wellbeing needs to incorporate the multiple dimensions that affect children’s lives. Bronfenbrenner’s ecosystem theory suggests that child development is a complex system of relationships influenced by multiple dimensions of the immediate environment, from the immediate home and school environment to a wide range of cultural values, laws and customs (Bronfenbrenner and Ceci, 1994; Bronfenbrenner and Morris, 1998). This theory argued that a child is influenced by five ecological forces: the microsystem, the mesosystem, the exosystem, the macrosystem, and the chronosystem. The microsystem is the most important level in ecological systems theory and contains the most immediate environment for developing children, such as peers, family, and school, who they live with or interact with, and the relationships they have with those people. The mesosystem is the interactions occurring in the child’s microsystem, such as the relationship between teachers and parents. The exosystem is a link between two settings that do not directly involve the child. The first one is the one in which individuals have an immediate role. The other is one where the person does not play an active role. Examples would be a father with his boss, peers with their parents, teachers with their principal, and the mass media. These are environments that the child is not involved in but nonetheless affect the child. For instance, parents may return home and be short tempered with their children because they are angry about things that happened in the workplace, which may have a negative impact on their children’s development. The macrosystem concentrates on how the cultural and social context influences all the others. The macrosystem is different from the previous three systems as it refers to the established society and culture in which the child is developing. The chronosystem consists of all environmental changes that occur in an individual’s life and their sociohistorical context. We suggest that the ecological systems theory provides a theoretical framework for exploring how reciprocal interactions between migrant children’s experiences and their environment during the COVID-19 have influenced their wellbeing. Among these the family and school setting is the most important. Figure 1 shows the analysis framework.

### 2.5 Present study: migrant children contextualization in Shanghai

In recent years, as the rural population continues to decline, more and more migrant workers and migrant children are moving to the cities. According to the seventh national population census in 2021, Shanghai had migrant population of 10.47 million, accounting for 42.1% of the total population. In the same year, there were around 1.17 million migrant children in Shanghai. As a city characterized by an aging population, 23.38% of which was composed of residents aged over 60 and 16.28% of residents aged over 65, and facing a decline in the birth rate, Shanghai is confronted by an urgent need to introduce migrant workers from other provinces in response to the negative effects of the aging population on its economic development (Shanghai Statistics Bureau, 2021).

In terms of migrant children’s education, migrant parents are required to obtain a Shanghai Residence Permit with at least 6 months social insurance in order for children to receive compulsory education. The residence permit system aims to manage and control people who do not have local hukou in urban areas. People can work and live legally in cities by applying for residence permits. Holders of residence permits enjoy the same rights as local residents such as education, health services and other social welfares. As migrant children grow up, they will reach a critical juncture of decision-making about their continuous studies in urban cities after compulsory education. Shanghai senior high school entrance examination policy is a selective examination aims in each city to sort out the students who have finished compulsory education and are qualified to receive senior secondary school education. Migrant children without a Shanghai household registration (hukou) or with less than 120 residence permit points cannot enroll in senior high school but to study in vocational schools after they finish junior high school (Shanghai Education Commission, 2019a,b, 2020). Those who have 120 points can study in general senior high school (Grade 10–Grade 12). Residence permit points are mainly composed of the applicant’s age, educational background, years of employment and social insurance contributions as well as professional and technical level, which makes it extremely difficult for most migrant workers. It is obvious that this system is talent-biased and wealth-biased. Some scholars have evaluated the extent of the friendliness of 16 cities in China and found that the senior high school entrance examination policy in Shanghai is the strictest one in the point-based admission system (Han et al., 2020). It reflected the unfriendliness of education toward migrant children in Shanghai, which can further impair the attractiveness of the city to migrant workers.

This study used Shanghai as a case study for the following reasons. Firstly, as an educated and economically developed city, Shanghai attracts a large number of migrant workers and their children to work and live compared to other cities. Secondly, compared to other cities, the combination of strict household registration policies (points for residence permits) and senior high school entrance examination policy in Shanghai create immense difficulties for migrant children’s future learning. These policies would give us a unique context that would help us gain insight into the wellbeing of migrant children during online classes under such policies.

Exenberger et al. (2019) found that children’s wellbeing deeply rooted in their culture of origin. Therefore, when discussing wellbeing and its indicators, the different cultural traditions of the East and the West should be considered. Numerous studies have been conducted on the experiences and wellbeing of students in different age groups in online leaning during the COVID-19 pandemic. However, migrant children in China are living in a different social and cultural environment compared to the general children. They suffered the most from the restrictions of household registration system (hukou), educational polices for migrant children and forced online schooling. Their struggles with structural inequalities were reinforced by online schooling during the pandemic. The forced online classes in China were a unique sample of online learning. This research expands upon the limited literature regarding the wellbeing of migrant children involved in online learning during the COVID-19 pandemic. Besides, what kind of impacts did the online learning have on the wellbeing of migrant children deserves to be analyzed by using Bronfenbrenner’s bioecological model. The bioecological model categorizes the environment into different levels and emphasizes the interrelationship between individuals and their surrounding environment. This may improve future online and blended education for students, families, and schools. It also provides unique insights into the means that schools can use to support students, parents and teachers as they deliver and engage with online schooling. Through this framework, we can also provide specific recommendations to improve their wellbeing. Previous research has only explored how online learning during COVID-19 influenced children’s wellbeing from a single respondent’s perspective. This study expands the perspective by including and combining the views of students, parents and teachers to explore the wellbeing of migrant children in online learning during COVID-19.

Accordingly, we have generated two research questions:

1. How has the online learning experience affected the wellbeing of migrant children in secondary school during the COVID-19 pandemic?
2. What can the experiences of migrant children help us learn about the role of adults in supporting migrant children’s wellbeing?

## 3 Materials and methods

### 3.1 Study design and setting

An intrinsic case study was adopted in this research to gather qualitative data, thereby helping the authors devote all their time and resources to a single case and develop an in-depth understanding of the research questions, focusing on the complexity and specificity of elements of that case, such as a community, school, family or organization, and telling a story about a bounded system, allowing the exploration of some complicated issues in that system (Denzin and Lincoln, 2005; Bryman, 2012; Johnson and Christensen, 2017). As almost all systems cover several components or parts, the exploration of how those parts or components work together is crucial to the understanding of the whole system.

The migrant population tends to be concentrated in the suburbs of Shanghai with low-cost housing and industrial areas where massive employment opportunities are provided (Bach, 2010; Han et al., 2020). Y district is an outer suburb of Shanghai and has attracted many migrant workers as it focuses on manufacturing, having a large number of factories. According to the 2020 national census, the population migrating to Y district increased to 108,453, an increase of 53.9% compared to the number in the 2010 national census. In Y district, the number of residents aged from 0 to 14 accounted for 9.2% of the district’s total population, a decrease of 0.3% compared with the number in the 2010 national census. The fieldwork was conducted from February to October 2020 in Shanghai S Public Junior High School (simply referred to as S School in the following section). The S school used to be a key public junior high school in its local district 12 years ago. Back then, it had few migrant children and no migrant children’s classes. However, many local students have transferred to urban schools since 2012, when a considerable number of migrant children began to enter S Public Junior High School, coupled with the demolition of local houses. Thus, the number of migrant children quickly exceeded the number of local students in this school. Despite the small number of local students remaining at S school, migrant children are still taught in separate classes from local children. Compared with the migrant children’s classes, the local students’ classes have a more intensive class schedule, especially for Grade 9 students who are going to take the senior high school entrance examination. Moreover, the teachers who have a wealth of teaching experience are arranged to teach the local children’s classes.

In early 2020, China adopted a series of nationwide prevention and control measures to address the spread of COVID-19. These measures include the implementation of community isolation, reinforced wearing of masks, the promotion of remote working and learning, and public gathering limits. In early February, Shanghai officially announced that schools would not open after the winter holidays. In order to ensure the safety of teachers and students, universities and schools in Shanghai started online education. Shanghai educators built a “classroom in the air” that made “suspension of classes without ceasing to teach, without ceasing to learn” a reality so that primary and secondary school students benefited a lot. The “classroom in the air” refers to the use of the internet and modern communication technology, broadcasting the teacher’s lectures in real time with video and audio etc., or recording the content of the teachers’ lectures and broadcasting it later, to transform the teaching process into a virtual one on the internet. There were no special requirements for the students, and as long as they had access to TV or internet. Students of each grade in Shanghai all follow the same timetable in their academic studies. The recorded video lessons could be played on television and various online platforms in accordance with the class schedule. There were 8 lessons each day, and each lesson lasted 40 min. The students could watch the recorded lessons together for the first 20 min, then the teachers and students in S school logged in the Tencent Meeting App, which allowed teachers to explain the content and answer questions raised by the students for the remaining 20 min. Online learning was suspended in April 2020 due to the temporary stability of the pandemic.

Since the COVID-19 restrictions had posed difficulties in speaking with the children, the fieldwork was conducted in two phases. The first phase consisted of online observations conducted from February to March 2020. The second phase consisted of semi-structured interviews held from April to October 2020 at S Junior High School. In the first phase, we looked at online learning from Grade 6 to Grade 9 to observe the activities of the teachers and students in the classes and how the students performed. The second phase provided an insight into how online education affected their wellbeing through interviews with the students, parents and teachers.

### 3.2 Participants and data collection

According to the qualitative data collected through observation and semi-structured interviews, migrant children at S school in Shanghai accounted for 80% of the total number of that school’s students. The participants included 31 migrant children (from Grade 6 to Grade 9), nine parents, 10 teachers and one school principal, all of whom were selected according to purposive sampling and maximum variation sampling. The participating students were between the ages of 12 and 15. As the first author worked in the Moral Education Department of S School and had the same educational experience as the migrant children, a good interpersonal relationship was established between the author and the students. The first author also relied on teachers as the intermediaries to connect her with the parents. When recruiting parents for the study, the author did not consider their occupations. The majority of them were factory workers, employed in the service sector, or small business or small factory owners, and a few of them worked as middle-level managers. Furthermore, some mothers of the migrant children were housewives. The highest education level of these parents was junior secondary school, and most of them had been living in Shanghai for over a decade. Most parents did not have 120 points for their residence permits.

Some of the interview questions used in the second phase were based on the observations of the online classes in the first phase. During the first phase of observations, it was seen that due to the limited time and tight schedules of the secondary school courses, the online class time was the same length as the class time would have been at school, requiring long hours on a computer or cell phone. The authors were not able to interact with everyone during the first phase of the online classes though, because they could not disturb the normal classroom order. However, through the online classes, some students and teachers came to have a certain understanding of the first author through the introduction provided by the school principal, which laid the foundation for entering the school in April 2020 during the second phase.

The first author gave participants information sheets and informed consents before they involved in this research. The informed consent forms were given to parents to decide whether their children attend this study. Migrant children themselves also agree to participate in the study after they have been informed all things that could their willingness to participate in research. The first author asked migrant children some questions during the interviews to construct and evaluate wellbeing. The interview questions include their learning difficulties, peer connections, how the pandemic affects their lives, interactions with teachers and peers, technical problems with equipment, and whether the home setting helped them to stay focused and engaged in online learning, and so forth. The two data collection techniques, observation and semi-structured interviews captured factors at each level of the ecological model. Observation can help us to observe the policy environment as a macrosystem, the online schooling as an exosystem, and some of the interactions between migrant children and other people in the microsystem. Through talking with children, parents, and teachers, the semi-structured interviews can help us gain insights into the connections among individuals within mesosystems and microsystems. In this research, we achieved reliability through discussion and consensus in order to understand the coded themes. Pseudonyms are used to protect the anonymity and confidentiality of participants. All ethical considerations, such as confidentiality, anonymity and informed consent were adhered to.

### 3.3 Data analysis

The interviews were conducted in Mandarin, audio-recorded and transcribed verbatim, each lasting from 1.5 to 2 h. Upon the completion of the two phases, Braun and Clarke (2006)’s six phases of thematic analysis were used to process the collected data. First, the first author read the transcripts in order to familiarize themselves with the data. NVivo 12 Plus was used to assist with coding. Second, all related codes were categorized into initial themes. After that, the first author grouped like codes together and thought about the features of these codes to find themes. Once themes were identified, the first author reviewed them and merged some themes when they had similar meanings. Next, themes were given names, and each theme was identified based on their meaning in the context of the data. All themes were refined and reviewed to ensure consistency by the second and third authors. As this research obtained data from different groups, data triangulation was employed to ensure the credibility of research results. Table 1 summarized main themes and sub-themes. Although the online observations presented a limitation to the study, the two phases did allow us to gather rich data regarding the views of the children, parents, and teachers. We report on our findings in the following section.

**Table 1:** Main themes from interviews.

Main themes	Sub-themes
Distress and helplessness of migrant children about online learning	Unable to adapt teaching method from high quality video-recorded lessons
	Poor academic performance after online class
Changes in emotion due to policies and online Learning	Different study progress when taking class with local students
	Lack of attention from school and policymakers
Less interaction between individuals and surroundings	Less interaction between migrant children and teachers
	Conflicts between parents and migrant children because of monitors
	Less interaction with peers
Cooperation between home and school to mitigate the negative effects on physical wellbeing	Eyestrain among migrant children
	Cooking lessons to alleviate the eyestrain

## 4 Results

Below we outline the main themes developed from our analysis, illustrated through brief extracts taken from the data.

### 4.1 Distress and helplessness of migrant children about online classes

The migrant children and teacher participants highlighted the benefits and drawbacks they perceived during the lockdown period schooling. Recorded video lessons in Shanghai for each subject were designed by outstanding teachers who had won national and municipal teaching competitions. Teachers believed that high quality video-recorded lessons could greatly help migrant children because the recorded lessons provide a valuable opportunity for them to learn from outstanding teachers. However, these high-quality video-recorded lessons had a limited impact and affect migrant children’s emotion because of time constraints.

Teachers in the downtown area record these courses, and they did a really good job—After all, they are experts at polishing their lessons in Shanghai, right? If they go and listen carefully, I think it will help the students a lot. However, the classroom in the air does not stop or wait for students to ask questions arising from the learning differences between students. The children in our suburbs still have a bit of a learning gap between them and those in the downtown area, so some of the migrant children were having difficulties listening to the class. Although the video can be played back, they may not be able to understand the lesson even if they watch it twice. Moreover, students have to do their homework at night and then upload it that day, so they do not have time to watch it again (Xin, Female, Grade 9 Math teacher and Head teacher from Migrant children’s class).

The high-quality video-recorded classes made migrant children feel distressed and helpless instead. The comments of two migrant children were typical:

I think it does not matter if he’s a special or an ordinary teacher, as long as he’s suitable for us, that’s the best, right? (Chai, Female, Grade 8 from Migrant children’s class).

We have been used to our own teacher’s teaching style. However, the teacher of the recorded class taught the English class in English the whole time. We did not understand it at all. I think the teachers on the television give me the personal feeling that they are condescending (Wen, Female, Grade 8 from Migrant children’s class).

One English teacher echoed students’ opinions:

The online lessons were helpful for our teachers. I am still using grammar lessons from video-recorded lessons for my students. However, we received feedback from our migrant children that they were unable to understand the full English online lesson. I felt sad that I had to spend 3 lessons to make the online lesson clear to my students (Mei, Female, Grade 8 English teacher from Migrant children’s class).

Before formal classes resumed, some parents believed that their children were not studying when watching computers or phones for long periods of time, which led to quarrels and eventually led to tragic suicides. Therefore, all schools in Shanghai postponed the school exams for 2 weeks to help students adjust to regular classes again in an attempt to ease the parents’ anxiety over the declining academic performance of students participating in online learning. In this case, after they came back to normal schooling, both migrant children and teachers found that most of the migrant students’ academic performance had dramatically declined during the period of online learning.

We were so miserable, with the sudden COVID-19 in our final year. The quality of online learning was bad, which caused a lot of students’ results to go down, which was quite annoying (Yu, Male, Grade 9 from Migrant children’s class).

There were some students in the local class whose grades went up by leaps and bounds. After listening to the solutions given by the teacher through the online classroom and combining them with their own teacher’s methods, they feel like they understood and had a good grasp of math, but some students in our migrant children’s class are like “bystanders,” their grades have dropped after more than 2 months, and they no longer know the basics (Xin, Female, Grade 9 Math teacher and Head teacher from Migrant children’s class).

### 4.2 Changes in emotion due to policies and online learning

It can be seen that the emotions of migrant children depend on factors within their specific context. It was found that the migrant children’s studies have been severely affected by the online learning context. The Grade-9 students who are going to take the high school entrance exam are the most affected ones.

Migrant children had been separated from local children in different classes at S School before online learning began. Since the beginning of the online learning, classes for migrant children and classes for local children have been combined. However, the progress and content of the lessons had varied greatly between the migrant children’s classes and the local students’ classes before the combined online classes began. Although the scores for the Physics, Chemistry, and PE exams are not counted in the senior high school entrance examinations in the case of migrant children, but they are required to take Physics and Chemistry final exams organized by S School. Although the above subjects are not counted in the total score for the examination, they are very important for future studies, and if migrant children have not been able to acquire enough knowledge related to the above subjects, further study will be difficult for them. Unlike the migrant children, the Grade-9 local students had learned all the new lessons about Physics and Chemistry before the outbreak of COVID-19, so they had more class time for other subjects. Tang and Suo are overachieving migrant children who were interested in Physics and Chemistry, but when their class was taught together with local students during the online learning period, they felt anxious and disturbed.

I’ve heard a lot of complaints from parents regarding chemistry classes, complaints that teachers do not pay enough attention to it, and the progress may not be the same. The main reason is that local students have too many class hours. They finished new classes before the winter holidays started. For the local class, the whole of the online learning period was spent on revision. For us, the entire period of the online classes was spent on new lessons, which means that we, the migrant children, have almost 4 months less time to revise for the Senior High School Entrance Examination. I went into an online meeting class for Chemistry one time. I found local students had all finished their second-round revision, but we have only just finished the new lessons (Tang, Male, Grade 9 from migrant children’s class).

… especially chemistry lessons, I cannot catch up and understand the recorded video lesson…my classmates are constantly absent from these courses. I think my Chemistry score has dropped drastically after attending the online course. I gave up completely. Teachers added extra lessons for local children in the evenings and weekends… the local students have more class hours than us (Suo, Male, Grade 9 from migrant children’s class).

Teachers of important subjects, such as Chinese, Mathematics and English, are also aware of this issue. With their time being limited, they cannot arrange more lessons for migrant children. Priority can only be given to local children, as they will take the senior high school entrance examination and then receive a high school education in order to be eligible for university entrance exams. Physics teachers and Chemistry teachers were also helpless in this situation.

We still have to favor the local students, who have to take the senior high school entrance exams. If the lesson is too easy, the local class will complain. But if I make it harder, the migrant children’s class will not be able to keep up, and it’s impossible to take care of all of that (Xin, Female, Grade 9 Math teacher and Head teacher from Migrant children’s class).

Their study progress is different from local students… “The people at the top (policymaker)” do not pay attention to it, so we certainly do not hold these two courses in high regard (Lu, Female, Grade 9 Chemistry Teacher).

### 4.3 Less interaction between individuals and surroundings

Online learning had caused a disruption in social interactions and developmental opportunities for migrant children. As mentioned before, the classes of local students and the classes of migrant children were merged, and classroom interactions between teachers and students were significantly reduced during online learning.

The teacher would often select students from the local class to answer questions and we would be at the back of the queue, and sometimes we would not get our turn (Yu, Female, Grade 9 from Migrant children’s class).

One teacher teaches two classes at the same time. It’s impossible to ask everyone to answer. It’s possible that not even one migrant child has been questioned during the whole week because there is only 20 min left for us after the video-recorded lesson (Xin, Female, Grade 9 Math teacher and Head teacher from Migrant children’s class).

Teachers did not grade our homework. Because they only graded local classes that they led. I did not know if I was right or wrong after I did my homework. I will not correct my homework if I do not hear it during the online class (Shu, Female, Grade 9 from Migrant children’s class).

In this circumstance, the migrant children have poor self-control ability and find it difficult to concentrate on a computer screen for a long time. In addition, children who use computers and mobile phones can easily be distracted by games or online social networking applications. Two migrant children and a teacher recall:

The efficiency of the online classes was very low. It felt refreshing to take an online course for the first time, but as time went by, my mind had already “flown away” (Yu, Female, Grade 9 from Migrant children’s class).

Many of us used our accounts to log onto a Tencent Meeting and pretended that we were in class. I split my screen so that I could play the game King of Glory, or chat with my friends on QQ (a Chinese instant messaging platform). When the teacher asked me to answer a question, I would pretend that the internet connection was having a problem, and I did not answer (Chai, Female, Grade 7 from Migrant children’s class).

Either students need parents who have to keep an eye on students the whole day, or you need students who are really self-motivated. But parents have to work and there are also few self-motivated students. The only student who made great progress during online schooling was because his mother, father and grandmother took turns sitting next to him to supervise his online schooling (Lu, Female, Grade 9 Chemistry Teacher).

The teachers and parents gradually became aware of this problem of low efficiency. In order to guide students and help them concentrate on learning, the teacher mentioned this matter to the parents in a WeChat group, asking parents to cooperate with the teacher to supervise the children’s study at home. Hence, some parents of migrant children have installed cameras in their children’s study rooms to monitor their behavior. This made many children feel uncomfortable and they resisted the action of their parents. One parent shared her story:

I saw other parents sharing pictures of their children’s learning taken by a camera (monitor) in WeChat Moments. I discussed this with my child: “We are not at home during the day, and no one will watch you. How about I install a monitor?” My daughter strongly disagreed with my idea. I said the camera just monitors her desk. She still disagreed. In spite of her disagreement, I installed the camera in her room. When I was not busy at work, I watched her through the camera. Sometimes when I saw her playing games on her mobile phone, I called her name. She was startled and immediately put the phone aside. A few days later, she moved the camera to another place and said: “You are so annoying” (Lin’s mother, Female, Grade 9 from Migrant children’s class).

Migrant children felt very nervous and uncomfortable with the surveillance. One migrant child recall:

Someone is monitoring me…it’s a camera. Our home has 4 cameras. Previously, my room was not monitored, and I motived myself to study, but they still set up one during the online learning period. I felt nervous and uncomfortable (Liang, Male, Grade 7 from Migrant children’s class).

Many parents of migrant children also believe that peer interaction stimulated by schools is also important for social development and well-being:

The online learning forced students to spend prolonged time at home, which restricted my daughter’s opportunities to develop her confidence when she is not with her family. In normal schooling, she can build connections with her peers. I’m worried about her happiness (Lin’s mother, Female, Grade 9 from Migrant children’s class).

I missed my classmates, but we were not allowed to meet up with each other because of the COVID-19. My friends were busy when I wanted to discuss math questions with them online. But if I’m in school, then I can discuss the problem directly with my peers in person (Jia, Female, Grade 9 from Migrant children’s class).

### 4.4 Cooperation between home and school to mitigate the negative effects on physical wellbeing

Children are often tired, especially after having stared at a screen all day long. Some migrant children were struggling to stare at the tiny screen of a phone from 8 a.m. to 6 p.m. Almost all migrant children and teachers mentioned their eyes were sore.

The online lessons are too long, my eyes are tired, and I want to close my eyes, and then I cannot listen anymore (Jing, Female, Grade 9 from Migrant children’s class).

Both teachers and student’s eyes are overwhelmed, especially those of some migrant children using mobile phones for the whole online learning period (Hong, Female, Grade 8 Head teacher from Migrant children’s class).

In order to alleviate the stress and improve the physical wellbeing of students, online learning introduced cooking classes at a later stage. That way, students could not only move their eyes move from the screen but could also learn new skills and achieve family-school interaction. The children of migrant families felt that this activity brought them closer to the teacher. One teacher shares his experience:

The teacher taught children how to cook in a video-recorded lesson. Our students showed their new skills to their parents at weekends. Some parents took photos and shared it in our class’s WeChat Group. Children were happy to share the cooking experience and their dishes with their parents and teachers. You know…children are always busy with study, and parents usually do not let them participate in housework. Some parents told me that they are happy to see their children learning to cook (Zhou, Female, Director of Teaching and Learning).

We were able to learn how to make cakes and tarts during the online learning period. I think baking has really helped alleviate some eyesight stress in my online class life. Although I did not bake well, I’m interested in it. Moreover, one of my friends lives next to my home. She went to vocational school after junior high school and studied baking. I was inspired by her and considered to study baking too (Su, Female, Grade 6 from Migrant children’s class).

## 5 Discussion

The COVID-19 pandemic-related interruption to education has had a profound effect on the educational system. Despite the fact that the findings of this research only apply to a small sample of migrant children, the data’s shared patterns point to important considerations regarding both how the online learning experience has affected the wellbeing of migrant children and what can be learned about rethinking secondary education in the wake of it. Bronfenbrenner’s bioecological perspective highlights how the migrant children’s wellbeing was affected by multiple external and internal factors, especially those stemming from the children’s school and family situation.

Firstly, during the COVID-19 lockdown period, all normal classes were suspended throughout Shanghai. The high quality of the video-recorded classes and online learning, which could be considered as exosystem according to Bronfenbrenner’s bioecological model, had a significant impact on the everyday learning of migrant children. While the video-recorded classes were of good quality, they put a heavier burden on migrant children than on the local children. The tight schedule of the daily online learning, teachers have no time to pay attention to students, students are not adapted to the way the teacher taught in the video recorded class, cannot get timely feedback from teachers, which ultimately led to the migrant children’s regressive performance after they returned to normal schooling. The classroom in the air takes away from the time students would normally spend with their own schoolteachers so that the teacher-student relationship between migrant children and their teachers is diminished, resulting in lower self-confidence, increased distress, helplessness and a lower sense of emotional wellbeing. This finding echoes the results of the studies by Bhamani et al. (2020) and Dong et al. (2020) who have found that children felt worried, irritable and anxious due to the lack of interaction, and delays in receiving substantive feedback from teachers about their schoolwork during the lockdown period. The emotional wellbeing of both Chinese migrant children and children from other countries was influenced by the exosystem, the mesosystem and the microsystem. Chinese migrant children, as a vulnerable group, deserve to be given even more attention regarding such experiences. Additionally, under the influence of the general environment surrounding COVID-19, using the high-quality video-recorded lessons instead of the traditional in-classroom lessons has changed the traditional teaching method. The emotional wellbeing of the Chinese migrant children is more seriously affected by decreased access to immediate help from schoolteachers, increased stress from studies, and the lower academic performance becoming apparent after they went back to normal schooling. This also provides valuable lessons for the future of online learning.

Secondly, before the local class and the classes of migrant children were merged, the migrant children did not feel angry because of the great inequality in their school treatment and the handling of their studies, but the online learning made the Grade 9 students realized that they were being treated differently. In addition, the forced neglect from teachers, and the lack of attention given to the education of migrant children by policymakers have led the migrant children to become aware of the difficulties they are encountering, and they have become anxious, angry and confused about their future, especially as they would have to take the senior high school entrance examination soon. The link between policymakers and schoolteachers is regarded as a mesosystem. Schoolteachers regarded the migrant children’s future education as being less important than that of local students. Therefore, they paid more attention to local students’ during online learning. During this process, the relationships between teachers and local students also formed a mesosystem, local students and migrant children formed a microsystem, and the situation made the migrant children directly and indirectly aware of an enormous inequality. These inequalities cause direct emotional problems (such as anxiety, helplessness and anger) for migrant children in online learning period and continued in normal schooling. Migrant children have no way to make any changes to the current situation, and they can only be forced to accept it. This finding is different from results from previous studies. Previous studies stressed that a teacher acts as an educator and mental health provider, not only needing to teach students knowledge, but also needing to promote the students’ wellbeing (Zins et al., 2007). The current research has found that policy makers and teachers have focused overwhelmingly on the local students’ academic achievements, thereby neglecting the mental health of the migrant students.

Regarding less interaction between individuals and surroundings, the data showed that teachers could not balance their teaching and interaction between local classes and migrant children’s classes. From the perspective of the microsystem, teachers paid less attention to migrant children, and migrant children also were affected by games or other mobile apps. In order to solve this problem, some parents installed monitors, which led to conflicts between the students and their parents. The continuity of the relationship with the teacher was seen as the key to their child’s engagement with school and overall wellbeing, highlighting the importance of the teacher–child relationship. In this study, it was found that migrant children have strong attachments to their class teacher. However, they have fewer interaction opportunities with their teacher compared to local students because most of the migrant children cannot take the senior high school entrance examination, which has resulted in an increased lack of attention being given to migrant children in online learning context. It is therefore difficult for these migrant students to get enough support from their schools and the community. A study found parents can support their children in learning (Zhang et al., 2020). Nevertheless, in this research, it was found that parents of migrant children as co-educators and regulators at home have to handle the pressures of work while home-schooling. Parents of migrant children are poorly educated, so they used monitoring cameras to replace their presence at home and take on the function of monitoring their children’s studies. Parents cannot teach their children to study, but the only thing they can do is to check the completion of homework and learning progress with the hope that children can achieve good results by themselves. Monitoring cameras were forcibly placed in the students’ rooms, so that the students had no privacy and felt uncomfortable. This result partially echoes the result of Brown et al. (2020) who have found it is difficult for parents with a low education level to replace the function of schoolteachers. But the difference is that although parents of migrant children are unable to contribute to mentoring, their high expectations of their children’s performance have led them to explore new ways (i.e., installing monitors to watch their children in real time) to make them believe that this assistance is beneficial to their children. Nevertheless, instead of keeping children in a healthy emotional condition during the pandemic, this action intensified the conflict between students and their parents. In addition, the long periods of online learning have diminished the opportunities for students to interact with each other. The extent of peer interactions was also regarded as an important factor in children’s wellbeing. Despite the existence of some negative impacts, for some children, established friendships and keeping in touch with friends online mitigated these negative impacts to a certain extent.

Regarding their physical wellbeing, 8 h of online classes a day made the migrant children tired, especially as some migrant children can only use their mobile phones to attend classes. Having to constantly look at things and read things on the small size mobile phone screen also made the students’ eyes tired. Previous studies also discovered that parents worried about their children’s eyesight because of 3–4 h of online learning (Christensen and Alexander, 2020; Zhao et al., 2020). Previous studies have only expressed parents’ concerns about their children’s eyesight wellbeing, but the present study provided a solution to this issue. For example, online cooking classes were introduced to the online learning schedule after several weeks of online learning in order to teaches students how to collaborate with their parents to cook together and display the results in the class WeChat group. This prompted the migrant children to take their eyes off their screens, relieving them to some extent of visual strain. Parents and teachers worked together as co-educators and educators to solve the children’s difficulties. Things like the cooking not only allowed students to relax from a stressful online class, but also provided time and space for parent–child relationships and strengthened the bond between school, home and student (Zhou et al., 2020).

## 6 Limitations and future research

This study has several limitations in this study that can be considered in future research. Firstly, it is worth noting that the one school investigated in this study cannot represent the whole of migrant children in China. Braun and Clarke (2012) argued that an important aspect of the qualitative paradigm is understanding the multiple meanings of small samples in the existing world, thereby generating contextual knowledge shaped by mainstream structures and processes. Thus, the experience gained from a small group of migrant children enables us to generate findings that may be able to be transferred to other contexts. Secondly, this study lack data from local parents. Comparing other factors between local and migrant parents that could give greater significance to this research, including parental involvement and supervision. Thirdly, this research discovered that migrant students perceived this discrimination or unequal treatment by teachers compared to local students during the online learning period and they felt this affect their social, emotional and physical wellbeing. Branscombe et al. (1999) and Zabala et al. (2020) found that the positive social identification of discriminated minorities can be a protective factor of wellbeing when facing discrimination. The rejection-identification model suggests that perceived racial prejudice has negative impact on individuals. However, a strong social identification with the own stigmatized group itself also alleviate negative impacts and promote wellbeing. As a vulnerable group, Chinese migrant children clearly understand that they cannot receive the same educational resources as locals. In future studies, it would be advisable to implement rejection-identification model and explore whether the social identification of Chinese migrant children can mitigate the negative effects of discrimination and promote their wellbeing.

Regarding its theoretical implications, this study advances the Bronfenbrenner bioecological model by testing its validity with a disadvantaged group in a non-western socio-cultural context. Regarding its long-term implications, firstly, the inequalities of education encountered by migrant children during the COVID-19 period have made them realize the disparities they have suffered in Shanghai. Findings of this study provide an empirical evidence base for future educational policy improvements, especially in supporting migrant children’s learning in urban areas. It is also important for future education reforms, including support for online learning, equal distribution of educational resources, and targeted teacher training. This reform can fundamentally improve the educational experience of underprivileged children. Regarding the broader impact, on the one hand, the results of the study may provide valuable insights and lessons for educators and policymakers facing similar challenges in other countries and regions. The case in Shanghai provides useful insights into how they might be better equipped to deal with similar situations. On the other hand, by exploring the roles of parents and schools during the online learning period, our research emphasized the importance of collaboration between families and schools. This may have positive impact in developing a more comprehensive family-school model. For example, the parents of migrant children often have problems with educating their children in the online learning period, i.e., long-term monitoring. Consequently, schools can establish a parents’ school to train the parents and establish incentive mechanisms to encourage parents to treat mental health issues seriously and correctly. Migrant children should not be forcibly monitored by their parents through the use of cameras during the online learning period, as such action tends to escalate the tension between parents and children and foster a rebellious psychology.

## 7 Conclusion

This study implemented Bronfenbrenner’s bioecological model to investigate emotional wellbeing, social wellbeing and the physical wellbeing of migrant children subject to the online learning experience during the COVID-19 period (Bronfenbrenner and Ceci, 1994; Bronfenbrenner and Morris, 1998). At the same time, this paper enables us to understand the roles that adults have played in the pandemic to support the wellbeing of migrant children. Our findings also provide lessons for parents, teachers and policymakers because of the discovery that the inequitable treatment of migrant children was exacerbated in the face of a public health emergency.

Firstly, the high-quality recorded video lessons put pressure on the migrant children due to the lessons containing only new material with no reviews. This makes it difficult for them to adapt to new learning methods, and test scores after they return to school showed that migrant children performed poorly in online learning. These problems caused distress and helplessness thereby affecting the emotional wellbeing of migrant children. Secondly, the differences in study progress between the migrant children and the local children that showed up during the online learning, and neglect from teachers and policymakers, made the migrant children anxious, angry and confused about their future. Similarly, this affected the emotional wellbeing of migrant children. Thirdly, the unequal treatment between local students and migrant children resulted in less interaction between migrant children and their surroundings, thereby affecting their social wellbeing. For example, the online learning reduced opportunities for migrant children to interact with their own teachers. Parents of migrant children are less educated and cannot assist their children with their homework. They want to use surveillance methods to support their children’s learning, but it had the opposite effect and simply provoked increased conflicts between children and their parents. In addition, the long period of online schooling decreases opportunities for migrant children to interact with their peers. When teachers focused on tutoring local students, migrant children had a greater need to interact with their peers in their studies. Finally, although the long hours of online lessons have affected the optical health of students, the subsequent additional cooking lessons have mitigated the optical health problem and strengthened the connections between home and school.

In this context, parents of migrant children as regulators and co-educators were not able to guide their children’s learning in the same way as other groups of parents did, but instead affected their children’s emotional wellbeing through the overwhelming use of monitors. In the initial phase of online schooling, teachers as educators and mental health supporters are not only unable to fulfill the students’ need to acquire knowledge, but also cannot promote the migrant children’s emotional wellbeing. However, in the later phase of the online learning, teachers acted as mental health providers, utilizing cooking classes to enhance the relationship between students and parents and provide opportunities for families to relax after a long period of intense academic pressure.

## Data availability statement

The raw data supporting the conclusions of this article will be made available by the authors, without undue reservation.

## Ethics statement

The studies involving humans were approved by the Hong Kong Polytechnic University, Departmental Research Committee (on behalf of Human Subjects Ethics Sub-Committee), Reference Number: HSEARS20200203003. The studies were conducted in accordance with the local legislation and institutional requirements. Written informed consent for participation in this study was provided by the participants’ legal guardians/next of kin.

## Author contributions

QD: Conceptualization, Data curation, Formal analysis, Investigation, Methodology, Project administration, Software, Writing – original draft, Writing – review & editing. QW: Project administration, Supervision, Validation, Writing – review & editing. QZ: Conceptualization, Validation, Writing – review & editing.
